# Season and vitamin D status are independently associated with glucose homeostasis in pregnancy

**DOI:** 10.1186/s12986-017-0203-5

**Published:** 2017-08-02

**Authors:** Eileen C. O’Brien, Elizabeth J. O’Sullivan, Mark T. Kilbane, Aisling A. Geraghty, Malachi J. McKenna, Fionnuala M. McAuliffe

**Affiliations:** 1 UCD Perinatal Research Centre, Obstetrics & Gynaecology, School of Medicine, National Maternity Hospital, University College Dublin, Dublin, Ireland; 2 Department of Clinical Chemistry, St Vincent’s University Hospital, Dublin, Ireland; 3 Department of Endocrinology, St Vincent’s University Hospital, Dublin, Ireland; 4 School of Medicine, University College Dublin, Dublin, Ireland

**Keywords:** Season, Vitamin D, 25OHD, Glucose metabolism, Insulin resistance, Circannual rhythm

## Abstract

**Background:**

Vitamin D status and season are intrinsically linked, and both have been proposed to be associated with glucose homeostasis in pregnancy, with conflicting results. We aimed to determine if exposure to winter and low maternal 25 hydroxyvitamin D (25OHD) in early pregnancy were associated with maternal glucose metabolism.

**Methods:**

This is a secondary data analysis of 334 pregnant women enrolled in the ROLO study, Dublin. Serum 25OHD, fasting glucose, insulin and insulin resistance (HOMA-IR) were measured in early (12 weeks’ gestation) and late pregnancy (28 weeks’ gestation). Season of first antenatal visit was categorised as extended winter (November–April) or extended summer (May–October). Multiple linear regression models, adjusted for confounders, were used for analysis.

**Results:**

Those who attended their first antenatal visit in extended winter had lower 25OHD compared to extended summer (32.9 nmol/L vs. 44.1 nmol/L, *P* < 0.001). Compared to those who attended their first antenatal visit during extended summer, extended winter was associated with increased HOMA-IR in early-pregnancy (46.7%) and late pregnancy (53.7%), independent of 25OHD <30 nmol/L and confounders. Early pregnancy 25OHD <30 nmol/L and extended winter were independently associated with significantly higher fasting glucose in late pregnancy (B = 0.15 and 0.13, respectively).

**Conclusions:**

Women who attended their first antenatal visit during the months of extended winter were more likely to have raised insulin resistance in early pregnancy, which had a lasting association to 28 weeks, and was independent of 25OHD. Our novel findings imply that seasonal variation in insulin resistance may not be fully explained by differences in vitamin D status. This could reflect circannual rhythm or seasonal lifestyle behaviours, and requires further exploration.

**Trial registration:**

ISRCTN registry, ISRCTN54392969, date of registration: 22/04/2009, retrospectively registered.

**Electronic supplementary material:**

The online version of this article (doi:10.1186/s12986-017-0203-5) contains supplementary material, which is available to authorized users.

## Background

Vitamin D status and season are intrinsically linked; upon exposure to natural ultraviolet solar radiation, endogenous skin production of vitamin D occurs. However, in countries at northern latitudes, such as Ireland (51–55°N), production reduces substantially from October to March [1, 2] with striking seasonal variation observed in 25-hydroxyvitamin D (25OHD) in pregnant and non-pregnant populations [3–5].

The presence of vitamin D receptors and expression of 1-α-hydroxylase enzyme in pancreatic beta cells suggest that vitamin D may be a modulator of insulin secretion and thus, influence glucose homeostasis [6]. In pregnancy, the association between low vitamin D levels and abnormalities in glucose tolerance is the subject of scientific debate, with some studies reporting that maternal insulin sensitivity, fasting glucose and gestational diabetes are not associated with 25OHD [7], while others have found inverse associations between 25OHD, fasting glucose [8] and risk of gestational diabetes [9]. However, the majority of vitamin D supplementation intervention trials in pregnancy have not had a significant impact on maternal and offspring health [10].

The physiological response to sunlight exposure, beyond that of vitamin D production, includes regulation of biological circadian and circannual rhythms, control of circulating melatonin, as well as production of nitric oxide, a vasodilator, and beta-endorphins which promote a feeling of well-being [11–13]. Circadian rhythms follow the daily, 24-h light-dark cycle [14], while seasonal fluctuations influence circannual rhythms [15]. Disruption of our circadian clock, for example though shift work that interferes with light-dark environmental phases, can negatively affect cardiometabolic function and increase insulin resistance [16]. The duration of natural sunlight in our environment varies depending on season, and although it is known that many animals adapt physiologically and behaviourally in order to survive the changing seasons, there is also evidence of circannual biological clocks among humans [15, 17]. Epidemiological data suggest that seasonality in sunlight and vitamin D are related to mortality and infection rates [18]. Seasonal variation has been reported in HbA_1c_ and glucose concentrations among individuals with type 2 diabetes, with highest levels observed in winter and lowest in summer [19–23]. Two separate cohort studies recently reported the prevalence of gestational diabetes diagnosis to be significantly higher in summer compared to winter [24, 25]. These findings are in keeping with another study that found the highest incidence of gestational diabetes in pregnancies with winter conception [26]. However, conflicting evidence exists [27, 28]. The seasonal differences outlined in the studies above could be attributed to seasonal 25OHD, or evolutionary circannual rhythms to protect mother and fetus. There are also other factors that are influenced by season: diet [29], physical activity levels [30], and even body mass index [31]. Due to the seasonal nature of 25OHD concentrations in many parts of the world [32], and the observed associations between vitamin D and insulin resistance, it would seem prudent to hypothesise that seasonal variation of glucose homeostasis may be mediated through 25OHD. In 2016 the Scientific Advisory Committee on Nutrition stated that the metabolic implications of seasonal variation in serum 25OHD remain unknown, highlighting the necessity for research on this topic [33].

We hypothesised that gestating through winter in early pregnancy is associated with both reduced serum 25OHD and increased markers of glucose metabolism (fasting glucose and insulin resistance); that reduced serum 25OHD is associated with increased markers of glucose metabolism; and that seasonal differences in these markers are associated with seasonal variation in serum 25OHD. Given the exploratory nature of this work, we also planned to conduct post-hoc exploratory analyses where appropriate.

## Methods

### Study design and setting

This is a secondary data analysis of 334 pregnant women enrolled in the ROLO (Randomised cOntrol trial of a LOw glycaemic index diet in pregnancy to prevent macrosomia) study between 2007 and 2011 at The National Maternity Hospital, Dublin, Ireland. Detailed methods and results of this study have been previously published [34, 35]. In brief, 800 secundigravida women, who had previously delivered a macrosomic infant (>4000 g), were randomised to receive either low glycaemic index dietary advice or usual care (no dietary advice) [34]. Although the primary outcome of birth weight did not significantly differ between the intervention and control group, the secondary outcomes of dietary glycaemic index, gestational weight gain and glucose intolerance were significantly reduced in the intervention arm. Insulin, but not HOMA, was significantly reduced in the intervention arm. No difference in serum 25OHD was noted between groups. The study was conducted per the guidelines laid down in the Declaration of Helsinki with institutional ethical approval and written maternal consent.

### Patient eligibility

Participants were recruited at the first antenatal hospital visit. Eligible participants were 10–18 weeks’ gestation, with a singleton pregnancy, over 18 years of age, not taking any medication, and without previous or current gestational diabetes. Only those who had completed the 2-year follow-up appointment had serum 25OHD measured in the pregnancy sample, thus 334 participants were included.

### Data collection

At the first antenatal consultation, participants were weighed in light clothing using a SECA weighing scales (SECA GmbH & Co. Kg. Germany) to the nearest 0.1 kg and height was measured without shoes to the nearest 0.1 cm using a wall-mounted stadiometer. Body mass index (BMI) (kg/m^2^) was calculated.

Food and beverages consumed over three consecutive days were recorded by participants during each trimester using 3-day food diaries. For this study, we analysed the early-pregnancy food diaries only. Dietary data from food diaries were analysed using NetWISP version 3.0 (Tinuviel Software, Llanfechell, Anglesey, UK). Participants also completed a questionnaire about use of dietary supplements. Information about the quantity of vitamin D within supplements was not collected. Emotional well-being was examined using the 5-item World Health Organization Well-Being Index (WHO-5) [36]. Physical activity, smoking and educational attainment were also self-reported.

Fasting serum blood samples were collected at the first antenatal visit (early pregnancy) and at the 28-week gestation visit (late pregnancy) for the measurement of insulin and 25OHD. Fasting blood glucose was measured at both time points, and a glucose challenge test (1-h post a 50-g glucose load) was performed at 28 weeks’ gestation.

### Laboratory analysis

Serum 25OHD concentrations were determined using the Elecsys Vitamin D Total (Roche Diagnostics GmbH, Mannheim, Germany) automated competitive binding protein assay [3]. The coefficients of variation (CV) for the 25OHD assay determined at assay verification were as follows: inter-assay CV, 8.9% at a concentration of 49.5 nmol/L and 3.7% at 103 nmol/L (intra-assay CV, 2.9% and 1.4% respectively). To ensure a high standard of analysis for serum 25OHD concentrations, the laboratory participates in an external quality assurance scheme; Vitamin D External Quality Assessment Scheme (DEQAS). Multianalyte profiling was performed on the Luminex Magpix system (Luminex Corporation Austin, USA). Plasma concentrations of insulin were determined by the Human Endocrine Panel. Maternal insulin resistance was calculated using the Homeostasis Model Assessment (HOMA-IR) equation: [fasting serum insulin (μU/ml) × fasting plasma glucose (mmol/l)/22.5].

### Statistical analysis

Serum 25OHD concentrations in early pregnancy were categorised according to the Institute of Medicine; <30 nmol/L and <50 nmol/L [37]. The change in 25OHD from early pregnancy to late pregnancy was calculated. Participants were dichotomised into the season during which the first antenatal visit took place; extended summer (May–October) or extended winter (November–April).

Data were assessed for normality using the Kolmogorov-Smirnov test and visual inspection of histograms. Non-normally distributed variables were log transformed prior to regression analysis. Maternal characteristics were explored using independent *t* tests, Mann-Whitney *U* tests and Chi-square tests. Simple linear regression was used to determine the univariate associations between metabolic markers, 25OHD, and early-pregnancy season. Multiple linear regression models were created using a forced-enter approach and were adjusted for confounders. To estimate the association between exposure to extended winter and the transformed metabolic markers, the reverse transformation of the B values from regression models were computed. Percentage differences in metabolic markers were calculated using the equation: ([e^B^-1]*100). We carried out sensitivity analysis using different thresholds for 25OHD in the multiple regression models. Statistical analyses were performed using IBM SPSS Statistics for Windows, Version 20.0 (IBM, Armonk, NY). Results were considered statistically significant if *P* < 0.05.

## Results

### Participant characteristics - demographics

Half of the participants attended their first antenatal visit during extended winter (November–April) (*n* = 166 [49.7%]). Maternal characteristics, as described in Table 1, did not differ between those who attended their first antenatal visit during extended winter or summer. A comparison of the maternal characteristics between those included (*n* = 334) and excluded from the original ROLO study is included in Additional file 1: Table S1.

**Table 1:** Participant Characteristics

		Season at first antenatal visit		
Characteristic	Total Group (*n* = 334)	Winter (*n* = 166)	Summer (*n* = 168)	*P* ^a^
Age (years)	33.0 (3.9)	32.8 (3.7)	33.2 (4.0)	0.409
Gestation at first antenatal visit (weeks)	12.8 (2.3)	12.7 (2.3)	12.9 (2.2)	0.556
BMI at first antenatal visit				
Mean (SD), (kg/m^2^)	26.3 (4.5)	26.3 (4.4)	26.2 (4.7)	0.948
Underweight (BMI <18.5 kg/m^2^) [n (%)]	2 (0.6)	1 (0.6)	1 (0.6)	0.953
Normal (BMI 18.5–24.9 kg/m^2^) [n (%)]	153 (45.8)	78 (46.4)	75 (45.2)	
Overweight (BMI 25.0–29.9 kg/m^2^) [n (%)]	125 (37.4)	60 (35.7)	65 (39.2)	
Obese (BMI >30.0 kg/m^2^) [n (%)]	52 (15.6)	27 (16.1)	25 (15.1)	
Smoking at baseline [n (%)]	7 (2.1)	4 (2.4)	3 (1.8)	0.697
Intervention group [n (%)]	171 (51.2)	84 (50.6)	87 (50.9)	0.829
Ethnicity: Caucasian [n (%)]	331 (99.1)	166 (100.0)	165 (98.2)	0.250
Achieved 3rd level education [n (%)]	177 (53.0)	87 (52.6)	90 (53.6)	0.940
Birth weight (g)	4065.9 (470.0)	4043.1 (434.4)	4088.4 (503.0)	0.382

Data are presented as mean (SD) unless otherwise stated

^a^Comparison between winter and summer

### Participant characteristics - 25OHD, season and markers of glucose metabolism

Median (IQR) 25OHD concentrations were 39.2 nmol/L (27.2 nmol/L) and 35.8 nmol/L (27.3 nmol/L) in early and late pregnancy, respectively. In early pregnancy, attending the first antenatal visit during extended winter was associated with significantly lower 25OHD concentrations than extended summer (32.9 nmol/L [22.6 nmol/L] vs. 44.1 nmol/L [27.6 nmol/L], *P* < 0.001). In early pregnancy, those who attended their first antenatal visit in extended winter were significantly more likely to be at risk of 25OHD deficiency (<30 nmol/L) and 25OHD inadequacy (<50 nmol/L), than those who attended their first antenatal visit in extended summer (Table 2). The median (IQR) change in 25OHD from early pregnancy to late pregnancy among women attending the first antenatal visit during extended winter was 0.60 nmol/L (22.42) and during extended summer was −11.14 nmol/L (22.75). Mean early pregnancy 25OHD versus month of first antenatal visit is displayed in Additional file 1: Fig. S1. In early pregnancy, insulin and HOMA-IR, but not glucose, were significantly higher among those who attended their first antenatal visit in extended winter (Table 2).

**Table 2:** Maternal 25OHD and markers of glucose metabolism in early and late pregnancy among the total group and categorised by season of first antenatal visit

		Season at first antenatal visit		
	Total Group (*n* = 334)	Winter (*n* = 166)	Summer (*n* = 168)	*P* ^d^
25OHD early <30 nmol/L ^a^	90 (26.9)	64 (38.6)	26 (15.5)	<0.001
25OHD early <50 nmol/L ^a^	205 (61.4)	121 (72.9)	84 (50.0)	0.002
25OHD 28 weeks <30 nmol/L ^a^	111 (33.2)	56 (33.7)	55 (32.7)	0.662
25OHD 28 weeks <50 nmol/L ^a^	211 (63.2)	103 (62.0)	107 (63.7)	0.999
HOMA-IR early ^b^	2.1 (2.6)	2.4 (2.8)	1.7 (2.0)	0.004
HOMA-IR 28 weeks ^b^	2.8 (3.15)	2.9 (3.4)	2.6 (2.6)	0.064
Insulin early (μU/mL) ^b^	11.3 (12.3)	12.8 (13.1)	9.3 (11.1)	0.002
Insulin 28 weeks (μU/mL) ^b^	14.4 (15.6)	14.6 (16.8)	14.1 (11.8)	0.108
Fasting glucose early (mmol/L) ^c^	4.48 (0.35)	4.47 (0.33)	4.49 (0.37)	0.566
Fasting glucose 28 weeks (mmol/L) ^c^	4.46 (0.46)	4.49 (0.45)	4.42 (0.46)	0.157
1-h GCT 28 weeks (mmol/L) ^c^	6.48 (1.44)	6.47 (1.39)	6.50 (1.48)	0.864

Data presented as ^a^n (%), ^b^median (interquartile range), ^c^mean (SD)

^d^Comparison between winter and summer

### Unadjusted analysis – 25OHD, season and markers of glucose metabolism

On unadjusted, simple linear regression, early-pregnancy 25OHD <30 nmol/L was not associated with any marker of glucose metabolism in early or late pregnancy (Table 3 [a]). Attendance at the first antenatal visit during extended winter, compared to summer, was associated with significantly higher early-pregnancy HOMA-IR (31.1%) and insulin (30.6%), but not glucose (Table 3 [b]).

**Table 3:** Unadjusted regression analysis: Percentage difference in markers of glucose metabolism associated with, (a) Early-pregnancy 25OHD < 30 nmol/L, and (b) first antenatal visit in extended winter

	(a) Early-pregnancy 25OHD <30 nmol/L				(b) First antenatal visit in extended winter			
Dependent Variable	% ∆^a^	B	95% CI	*P*	% ∆^a^	B	95% CI	*P*
HOMA-IR early	18.5%	0.17	(−0.04, 0.39)	0.110	31.1%	0.27	(0.08, 0.46)	0.006
HOMA-IR 28 weeks	11.6%	0.11	(−0.13, 0.39)	0.371	21.9%	0.20	(0.00, 0.40)	0.051
Insulin early (μU/mL)	16.2%	0.15	(−0.06, 0.35)	0.173	30.6%	0.27	(0.08, 0.45)	0.005
Insulin 28 weeks (μU/mL)	7.3%	0.07	(−0.16, 0.30)	0.528	19.2%	0.18	(−0.02, 0.37)	0.074
Fasting glucose early (mmol/L)		0.03	(−0.06, 0.12)	0.541		−0.02	(−0.09, 0.05)	0.566
Fasting glucose 28 weeks (mmol/L)		0.09	(−0.02, 0.21)	0.112		0.07	(−0.03, 0.17)	0.157
1-h GCT 28 weeks (mmol/L)		0.04	(−0.32, 0.41)	0.824		−0.03	(−0.34, 0.29)	0.864

^a^ % difference in metabolic marker

### Adjusted analysis (model 1) – 25OHD, season and markers of glucose metabolism

Multiple linear regression models (Table 4) included both 25OHD <30 nmol/L and extended winter as predictors of the metabolic markers (Model 1), and were also adjusted for confounders (Model 2). Prior to analysis, collinearity between the two predictor variables was measured using Spearman’s rho to ensure they were independent; relatively low correlation was observed between the variables (*r* = 0.24, *P* < 0.001). Attendance at the first antenatal visit in extended winter, compared to summer, was associated with significantly higher HOMA-IR (32.8%) and insulin (32.4%) in early pregnancy, independent of 25OHD <30 nmol/L. Neither extended winter nor 25OHD <30 nmol/L were associated with glucose in early or late pregnancy, or the GCT in late pregnancy (Model 1, Table 4). An interaction term (early-pregnancy 25OHD <30 nmol/L * winter) was subsequently added to the model, but was not significant in any model (data not shown).

**Table 4:** Multiple Linear Regression analysis and associated percentage difference in markers of glucose metabolism (Model 1 unadjusted, and Model 2 adjusted)

		Model 1 – Unadjusted model					Model 2 – Final Adjusted model				
Dependent Variable	Independent variable (early pregnancy)	% ∆^a^	B	95% CI	*P*	Model *P*	% ∆^a^	B	95% CI	*P*	Model *P*
HOMA-IR early	Winter	32.8%	0.28	(0.08, 0.49)	0.008	0.008	46.72%	0.38	(0.15, 0.62)	0.001	<0.001
	25OHD <30 nmol/L	11.6%	0.11	(−0.11, 0.32)	0.325		11.64%	0.11	(−0.14, 0.36)	0.382	
HOMA-IR 28 weeks’	Winter	29.7%	0.26	(0.04, 0.49)	0.024	0.051	53.70%	0.43	(0.17, 0.69)	0.002	0.001
	25OHD <30 nmol/L	4.1%	0.04	(−0.20, 0.28)	0.753		3.00%	0.03	(−0.25, 0.31)	0.837	
Insulin early (μU/mL)	Winter	32.4%	0.28	(0.08, 0.48)	0.007	0.010	47.57%	0.39	(0.16, 0.62)	0.001	<0.001
	25OHD <30 nmol/L	8.3%	0.08	(−0.14, 0.29)	0.474		8.16%	0.08	(−0.17, 0.32)	0.530	
Insulin 28 weeks (μU/mL)	Winter	25.9%	0.23	(0.01, 0.46)	0.041	0.102	49.53%	0.40	(0.14, 0.66)	0.003	0.002
	25OHD <30 nmol/L	1.0%	0.01	(−0.22, 0.24)	0.927		−0.17%	0.00	(−0.28, 0.28)	0.991	
Fasting glucose early (mmol/L)	Winter		−0.02	(−0.11, 0.06)	0.583	0.713		−0.04	(−0.13, 0.06)	0.435	0.021
	25OHD <30 nmol/L		0.03	(−0.06, 0.13)	0.469			0.06	(−0.04, 0.16)	0.257	
Fasting glucose 28 weeks (mmol/L)	Winter		0.07	(−0.04, 0.18)	0.185	0.118		0.13	(0.00, 0.26)	0.045	0.015
	25OHD <30 nmol/L		0.08	(−0.04, 0.19)	0.208			0.15	(0.01, 0.29)	0.041	
1-h GCT 28 weeks (mmol/L)	Winter		0.04	(−0.31, 0.39)	0.806	0.946		0.02	(−0.41, 0.45)	0.930	0.688
	25OHD <30 nmol/L		0.03	(−0.35, 0.41)	0.873			0.02	(−0.45, 0.50)	0.923	

^a^ % difference in metabolic marker

Model 1: model not controlled for confounders. Model 2: model controlled for BMI (kg/m^2^), education > secondary school, being part of the ROLO study intervention group, early-pregnancy energy (kcal), early-pregnancy protein (g), early-pregnancy fat (g), moderate-vigorous activity, well-being score and dietary supplements

### Post-hoc analysis - exploring potential confounders

Dietary intakes, lifestyle behaviours, and emotional well-being were analysed by season of first antenatal visit **(**Additional file 1: Table S2). Attendance at the first antenatal visit during extended winter was associated with reduced intake of energy (kcal) (winter vs. summer; 1795.2 ± 412.0 vs. 1904.7 ± 418.2, *P* = 0.031), protein (g) (winter vs. summer; 74.4 ± 17.4 vs 79.7 ± 20.7, *P* = 0.023) and fat (g) (winter vs. summer; 69.9 ± 19.7 vs 77.2 ± 22.8, *P* = 0.005). Physical activity and emotional well-being scores were similar between extended winter and extended summer groups.

### Adjusted analysis (model 2) – 25OHD, season and markers of glucose metabolism

The final adjusted, multiple linear regression model included extended winter at first antenatal visit, 25OHD <30 nmol/L, and the following confounders: BMI (kg/m^2^), education, being part of the ROLO study intervention group, early-pregnancy energy (kcal), protein (g) and fat (g), physical activity and well-being score (Model 2, Table 4). Compared to those who attended their first antenatal visit during extended summer, extended winter was associated with increased early-pregnancy HOMA-IR (46.7%) and insulin (47.6%), independent of 25OHD <30 nmol/L and confounders. The early-pregnancy exposure to extended winter, compared to extended summer, was also associated with increased HOMA-IR (53.7%) and insulin (49.5%) in late pregnancy. Early-pregnancy 25OHD <30 nmol/L and exposure to extended winter were independently associated with significantly higher glucose in late pregnancy (B = 0.15, and 0.13, respectively), controlling for confounders.

### Sensitivity analysis

There were no significant changes in the multiple regression models using different 25OHD variables as independent variables associated with markers of glucose metabolism; early pregnancy 25OHD (continuous); greater or less than 50 nmol/L (insufficiency), greater or less than 39.2 nmol/L (median) (data not shown). Model 2 of the multiple linear regression analysis was additionally controlled for change in 25OHD from early to late pregnancy **(**Additional file 1: Table S3). An increase in 25OHD from early to late pregnancy was not associated with markers of glucose metabolism.

## Discussion

Exposure to extended winter in early pregnancy is associated with increased insulin resistance in early and late pregnancy, and increased fasting glucose in late pregnancy, independent of 25OHD status. Additionally, those at risk of vitamin D deficiency in early pregnancy are more likely to have increased fasting glucose in late pregnancy, independent of season. Given that the association between early gestation through extended winter and insulin resistance is independent of serum 25OHD <30 nmol/L, our novel findings imply that seasonal variation in insulin resistance may not be fully explained by differences in vitamin D status. Furthermore, no interaction effect was observed between season and vitamin D with regards to glucose homeostasis. Additionally, lifestyle behaviours that may be influenced by seasonal changes (dietary intakes, physical activity, and well-being) did not explain the associations between exposure to winter at the first antenatal visit and markers of glucose metabolism. Although the seasonal variation observed in insulin resistance may be caused by unknown seasonal behaviours or residual confounding factors that we have not included in this analysis, it is possible that there is an unequivocal seasonal cause or circannual rhythm associated with increased insulin resistance in early pregnancy during winter months. Regardless of the underlying cause for increased markers of glucose metabolism, our findings suggest that during early pregnancy, exposure to a typical winter in the northern hemisphere, which often includes low serum 25OHD, is unfavourable for maternal glucose homeostasis.

### Circannual rhythms and glucose homeostasis

Circannual rhythms have been researched in animals with regards to reproduction, migration and hibernation, and are biological survival mechanisms to allow certain events to occur at the most favourable time in the year [38]. Humans also evolved to adapt to environmental daily and seasonal changes, with a central biological clock located in the suprachiasmatic nucleus of the hypothalamus, responsible for timing of metabolism, physiology and behaviours [17]. During hunter-gatherer times, summer was a season of increased daylight and nutritional abundance that prepared our ancestors for harsher winter conditions by depositing fat reserves during autumn [17]. Through technological advances, humans have access to light continuously and are no longer dependant on the rising and setting of the sun to regulate circannual rhythms. However, circadian clock genes that respond to light are still part of our genome [39]. Melatonin, a pineal hormone regulated by light exposure, peaks during night after the onset of sleep, with little production during daylight hours [13]. Melatonin inhibits the release of insulin from pancreatic beta cells [40, 41], although conflicting research exists with the Nurses’ Health Study finding low melatonin associated with increased risk of type 2 diabetes, potentially medicated through increased insulin resistance [42]. It may be hypothesised that during winter months, which are characterised by increased darkness, women may be exposed to melatonin close to morning and evening meals, reducing their ability to deal with carbohydrate based foods. It is also possible that the milieu of higher melatonin and insulin resistance during winter in early pregnancy may set the scene for glucose homeostasis throughout the remaining months of pregnancy. This could explain why our findings of exposure to extended winter in early pregnancy is associated with increased HOMA and fasting glucose at 28 weeks’ gestation, although research is needed to explain this potential hypothesis.

The phenomenon of circannual rhythms has also been explored in relation to gestational diabetes [24–26, 28]. Recently, Moses et al. prospectively evaluated 7369 oral glucose tolerance tests (OGTT) over a 3-year period in Australia [24]. The prevalence of gestational diabetes diagnosed on 1-h OGTT result was 45% higher in November (summer) and 46% lower in July (winter) than the overall prevalence, however, diagnoses based on fasting glucose concentrations were similar across the year. The OGTTs were carried out at 24–28 weeks’ gestation; thus, summer at the time of OGTT would correspond approximately with winter in early pregnancy in our population. Similarly, in Sweden, Katsarou et al. analysed 11,538 pregnancies, and found a 51% increase in gestational diabetes diagnosed by OGTT in the months of June to July (summer), compared to other months [25]. Verburg et al. reported that conception during winter was associated with a significantly higher incidence of gestational diabetes than during summer [26]. We carried out glucose challenge tests (1-h post 50 g glucose load) for all women in our population, not OGTT as in the above studies. Exposure to winter in early pregnancy was not associated with glucose challenge test results, however it was associated with increased fasting glucose and insulin resistance at 28 weeks. Conversely, Janghorbani et al. did not find an association between season and gestational diabetes incidence among 4942 British women between 1996 and 1997 [28].

In non-pregnant adult populations, conflicting results have been reported regarding seasonal variation HbA_1c_ (a measure that reflects average circulating blood glucose concentrations for the previous 90 days) and glucose concentrations among individuals with type 2 diabetes [19, 43]. Carney et al. observed significantly higher HbA_1c_ in the UK among female patients with type 2 diabetes in the months February, March and June, and suggested that poorer diet and reduced physical activity associated with Christmas and Easter celebrations may be involved [20]. Fasting glucose and HbA_1c_ were lowest among Greek patients with type 2 diabetes during August and September [23]. The authors suggested that a combination of seasonal changes to an individuals’ physical activity, diet, sunlight exposure, weather pattern, psychological variables, and weight changes may be contributing factors [23]. In our cohort, dietary energy, protein and fat were significantly higher during extended summer, while carbohydrates, glycaemic index, glycaemic load, physical activity and well-being were no different across seasons. During summer, early pregnancy energy (kcal) was associated with a negligible reduction in late pregnancy insulin resistance (−0.1%), while fat (g) was associated with a small increase in late pregnancy insulin resistance (1.4%). The other seasonal behaviours were not significantly associated with metabolic markers.

Traditionally, and in may regions of the world today, seasonal availability of food determines dietary intakes. However, in developed countries, seasonal availablity of food has a limited impact on diet, although seasonal differences in dietary intakes among pregnant women have been observed [29]. It is possible that the increased energy, protein and fat observed in our study during extended summer could be due to changes in dietary habits during the summer holidays. Debate also exists in relation to seasonality of physical activity levels [30, 44]. Our study did not find a difference in self-reported physical activity across the seasons.

Whether environmentally or biologically driven, we found novel evidence of seasonal rhythms in insulin resistance. It could be hypothesised that increased insulin resistance during winter pregnancies is an evolutionary protective mechanism for the mother and fetus, of laying down additional fat stores to ensure survival through winter. Further research is required to determine the physiological pathways and clinical significance of our observations.

### 25OHD and glucose homeostasis

Within our cohort, early-pregnancy 25OHD <30 nmol/L was associated with significantly higher fasting glucose at 28 weeks’ gestation, independent of season and potential confounders. Low 25OHD at the first antenatal visit was not associated with insulin resistance in early or late pregnancy when controlling for season and confounders. Our results are similar to those of the HAPO trial, which found that maternal insulin sensitivity and gestational diabetes were not associated with 25OHD [7]. However, unlike the HAPO trial, we found increased fasting plasma glucose in late pregnancy among those at risk of vitamin D deficiency. A recent meta-analysis of observational studies reported that vitamin D < 50 nmol/L was associated with an increased risk (OR = 1.53) of developing gestational diabetes [9].

Many women who gestate through a typical winter in the northern hemisphere are at risk of vitamin D deficiency [4, 5]. Poor vitamin D intakes have been reported among pregnant women living in Ireland [5]. In the clinical setting, pregnant women should receive advice about vitamin D, especially during winter. Winter-time supplementation of 1000 IU/day vitamin D is an effective strategy to improve 25OHD in pregnancy [45]. At present, there remains a lack of consensus regarding recommendations for UVB exposure for the production of vitamin D for pregnant women [46]. Our findings suggest that women at risk of vitamin D deficiency in early pregnancy have altered glucose metabolism in later gestation, but the evidence is not consistent for insulin resistance. Thus, recommendations for vitamin D supplementation to improve glucose homeostasis in pregnancy cannot be made based on our results.

### Strengths and limitations

A potential limitation of our work is the dichotomised seasonal divisions. However, we chose these for multiple reasons; the HAPO study used similar divisions in countries with a latitude of 41.9–43.7° North [7], mean daily temperatures in Ireland are highest during the months May to October [47], and we based these seasons on 25OHD concentrations for the month of the first antenatal visit (Additional file 1: Fig. S1). Some studies have attributed seasonal differences in gestational diabetes to temperature changes. Although we did not collect data on daily temperature, the temperatures in Ireland are relatively stable across the year, with mean temperature of 5.3 °C in January and 15.6 °C in July [47], reducing the likelihood that temperature is responsible. Detailed data on micronutrient supplement use were not available, however, use of dietary supplements (yes/no) was included as a confounder in multiple regression models. Data relating to pre-pregnancy lifestyle behaviours were not collected. The generalisability of our findings may be limited, as participants had previously given birth to a macrosomic infant (>4000 g), and half the women received advice to follow a low glycaemic diet. We controlled for intervention grouping in the multiple linear regression models. Furthermore, only women who attended the 2-year follow-up appointment had pregnancy serum 25OHD measured, and some differences were observed in the baseline characteristics of those included and excluded from this analysis [48]. Our research would be strengthened with the addition of HbA_1c_ data in pregnancy and additional maternal lifestyle variables to allow the associations to be teased out. The use of GTT (75 g glucose load), rather than glucose challenge test, would have allowed for better comparison with the seasonal gestational diabetes papers discussed, however universal screening for gestational diabetes using GTT is not currently standard practice in Ireland. Additionally, our findings are observational in nature thus we cannot determine causality.

## Conclusions

Women who attend their first antenatal visit during the months of extended winter were more likely to have raised insulin resistance in early and late pregnancy, and increased fasting glucose in late pregnancy, independent of 25OHD status. Participants at risk of vitamin D deficiency at their first antenatal visit were likely to have increased fasting glucose at 28 weeks’ gestation, regardless of season. While no interaction effect was observed between season and vitamin D with regards to glucose homeostasis, it is well recognised that 25OHD declines in winter, and is a modifiable factor. Our novel finding of the association between season and insulin resistance, independent of 25OHD, could reflect circannual rhythm or seasonal lifestyle behaviours and requires further exploration.

## Additional file

Additional file 1: Figure S1. Mean early pregnancy 25OHD (nmol/L) versus month of first antenatal visit. **Table S1.** Maternal characteristics between included and excluded ROLO Study participant. **Table S2**. Dietary intakes, emotional well-being and lifestyle behaviours according to season of first antenatal visit. **Table S3**. Multiple Linear Regression analysis and associated percentage difference in markers of glucose metabolism (Model 2 additionally adjusted for change in 25OHD from early to late pregnancy). (DOCX 37 kb)

## Abbreviations

25OHD: 25 hydroxyvitamin D
BMI: Body mass index
HOMA-IR: Homeostasis Model Assessment for insulin resisitance
