# Supplementary material for: Season and vitamin D status are independently associated with glucose homeostasis in pregnancy
Source: Nutr Metab (Lond). 2017 Aug 2;14:50. doi: 10.1186/s12986-017-0203-5 (PMC5539754; doi:10.1186/s12986-017-0203-5)
Supplement: Additional file 1: Figure S1. — Mean early pregnancy 25OHD (nmol/L) versus month of first antenatal visit. Table S1. Maternal characteristics between included and excluded ROLO Study participant. Table S2. Dietary intakes, emotional well-being and lifestyle behaviours according to season of first antenatal visit. Table S3. Multiple Linear Regression analysis and associated percentage difference in markers of glucose metabolism (Model 2 additionally adjusted for change in 25OHD from early to late pregnancy). (DOCX 37 kb) [file 12986_2017_203_MOESM1_ESM.docx]

Additional file 1

Figure S1. Mean early pregnancy 25OHD (nmol/L) versus month of first antenatal visit


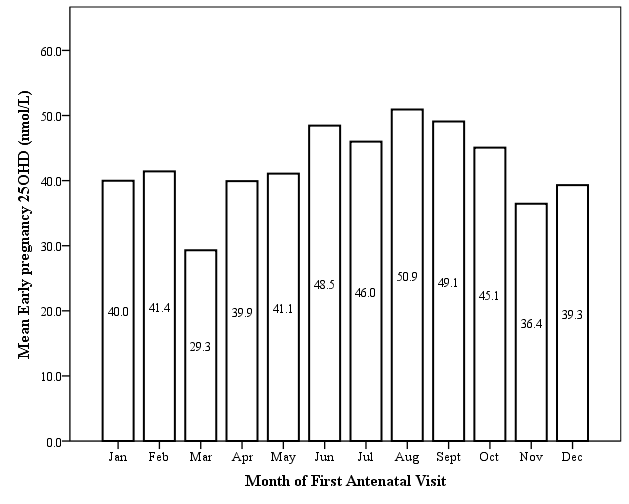


Table S1. Maternal characteristics between included and excluded ROLO Study participant

|  | Included (n = 334) | Excluded (n = 419) | *P* |
| --- | --- | --- | --- |
| Age (years) ^a^ | 33.02 (3.84) | 32.01 (4.44) | 0.001 |
| Weight at first antenatal visit (kg) ^a^ | 72.79 (13.33) | 74.08 (14.58) | 0.210 |
| BMI at first antenatal visit (kg/m^2^) ^a^ | 26.26 (4.54) | 27.04 (5.18) | 0.030 |
| Smoking at baseline [n (%)] ^b^ | 7 (2.10%) | 23 (5.41%) | 0.032 |
| Intervention group [n (%)] ^b^ | 170 (50.90%) | 197 (46.35%) | 0.242 |
| Achieved 3^rd^ level education [n (%)] ^b^ | 177 (59.60%) | 171 (52.13%) | 0.073 |
| Ethnicity: white Irish [n (%)] ^b^ | 331 (99.10%) | 411 (97.16%) | 0.246 |
| Fasting glucose early (mmol/L) ^a^ | 4.48 (0.35) | 4.44 (0.39) | 0.235 |
| Fasting glucose 28 weeks (mmol/L) ^a^ | 4.46 (0.46) | 4.51 (0.65) | 0.240 |
| 1-hour GCT 28 weeks (mmol/L) ^a^ | 6.49 (1.44) | 6.68 (1.68) | 0.094 |
| Insulin early (µU/mL) ^c^ | 11.32 (12.29) | 12.29 (12.37) | 0.410 |
| Insulin 28 weeks (µU/mL) ^c^ | 14.45 (15.58) | 15.06 (14.42) | 0.523 |
| HOMA-IR early ^c^ | 2.12 (2.54) | 2.22 (2.35) | 0.690 |
| HOMA-IR 28 weeks ^c^ | 2.80 (3.15) | 2.91 (2.93) | 0.980 |
| Data presented as: ^a^ mean (SD), t-test; ^b^ n (%), chi-square; ^c^ median (interquartile range), Mann-Whitney U | | | |

Table S2. Dietary intakes, emotional well-being and lifestyle behaviours according to season of first antenatal visit

|  |  | Season of first antenatal visit | |  |
| --- | --- | --- | --- | --- |
|  | Total Group | Winter | Summer | *P ^c^* |
| Energy (kcal) ^a^ | 1850.5 (417.3) | 1795.2 (412.0) | 1904.7 (418.2) | 0.031 |
| Protein (g) ^a^ | 77.0 (19.2) | 74.4 (17.4) | 79.7 (20.7) | 0.023 |
| Protein as percentage energy (%) ^a^ | 16.8 (3.0) | 16.8 (3.1) | 16.8 (2.9) | 0.895 |
| Carbohydrate (g) ^a^ | 233.8 (60.8) | 230.9 (64.8) | 236.6 (56.9) | 0.442 |
| Carbohydrate as percentage energy (%) ^a^ | 50.6 (6.7) | 51.2 (6.5) | 49.9 (6.9) | 0.120 |
| Total fat (g) ^a^ | 73.6 (21.5) | 69.9 (19.7) | 77.2 (22.8) | 0.005 |
| Total fat as percentage energy (%) ^a^ | 35.6 (5.8) | 35.0 (5.7) | 36.2 (5.7) | 0.088 |
| Glycaemic Load ^a^ | 136.0 (35.9) | 134.6 (39.9) | 137.4 (31.5) | 0.533 |
| Glycaemic Index ^a^ | 57.5 (4.0) | 57.8 (3.9) | 57.2 (4.0) | 0.174 |
| WHO-5 Wellbeing Percentage ^a^ | 57.9 (14.5) | 58.1 (14.4) | 57.6 (14.7) | 0.784 |
| Number of days with ≥30 minutes walking ^b^ | 3.6 (1.8) | 3.6 (1.8) | 3.5 (1.9) | 0.837 |
| Total minutes physical activity/week ^b^ | 100.0 (80.0) | 100.0 (80.0) | 100.0 (80.0) | 0.728 |
| Total minutes moderate - vigorous physical activity/week ^b^ | 20.0 (60.0) | 20.0 (60.0) | 40.0 (60.0) | 0.439 |
| Hours/day watching television ^b^ | 2.0 (1.0) | 2.0 (1.0) | 2.0 (1.0) | 0.111 |
| Data presented as ^a^ mean (SD), ^b^ median (interquartile range)  ^c^ Comparison between winter and summer | | | | |

Table S3. Multiple Linear Regression analysis and associated percentage difference in markers of glucose metabolism (Model 2 additionally adjusted for change in 25OHD from early to late pregnancy)

| Dependent Variable | Independent variable | %∆^a^ | B | 95% CI | *P* | Model *P* |
| --- | --- | --- | --- | --- | --- | --- |
| HOMA-IR early | Winter (early) | 37.00% | 0.31 | (0.05, 0.58) | 0.018 | 0.001 |
|  | 25OHD <30nmol/L (early) | 7.24% | 0.07 | (-0.20, 0.34) | 0.610 |  |
|  | Change in 25OHD | 0.62% | 0.01 | (0.00, 0.01) | 0.088 |  |
| HOMA-IR 28 weeks’ | Winter (early) | 38.39% | 0.32 | (0.05, 0.60) | 0.019 | 0.002 |
|  | 25OHD <30nmol/L (early) | -3.69% | -0.04 | (-0.32, 0.24) | 0.791 |  |
|  | Change in 25OHD | 0.68% | 0.01 | (0.00, 0.01) | 0.067 |  |
| Insulin early (µU/mL) | Winter (early) | 38.00% | 0.32 | (0.07, 0.58) | 0.014 | 0.001 |
|  | 25OHD <30nmol/L (early) | 4.06% | 0.04 | (-0.23, 0.31) | 0.770 |  |
|  | Change in 25OHD | 0.67% | 0.01 | (0.00, 0.01) | 0.061 |  |
| Insulin 28 weeks (µU/mL) | Winter (early) | 34.45% | 0.30 | (0.03, 0.56) | 0.028 | 0.003 |
|  | 25OHD <30nmol/L (early) | -5.93% | -0.06 | (-0.34, 0.21) | 0.661 |  |
|  | Change in 25OHD | 0.68% | 0.01 | (0.00, 0.01) | 0.059 |  |
| Fasting glucose early (mmol/L) | Winter (early) |  | -0.05 | (-0.15, 0.05) | 0.355 | 0.109 |
|  | 25OHD <30nmol/L (early) |  | 0.04 | (-0.06, 0.15) | 0.414 |  |
|  | Change in 25OHD |  | 0.00 | (0.00, 0.00) | 0.693 |  |
| Fasting glucose 28 weeks (mmol/L) | Winter (early) |  | 0.13 | (-0.01, 0.27) | 0.071 | 0.053 |
|  | 25OHD <30nmol/L (early) |  | 0.14 | (-0.01, 0.29) | 0.063 |  |
|  | Change in 25OHD |  | 0.00 | (0.00, 0.00) | 0.587 |  |
| 1-hour GCT 28 weeks (mmol/L) | Winter (early) |  | -0.01 | (-0.50, 0.47) | 0.961 | 0.486 |
|  | 25OHD <30nmol/L (early) |  | 0.11 | (-0.41, 0.62) | 0.682 |  |
|  | Change in 25OHD |  | 0.00 | (-0.01, 0.02) | 0.672 |  |
| a % difference in metabolic marker  Model controlled for BMI (kg/m^2^), education>secondary school, being part of the ROLO study intervention group, early-pregnancy energy (kcal), early-pregnancy protein (g), early-pregnancy fat (g), moderate-vigorous activity, well-being score and dietary supplements. | | | | | | |
